# Efficacy of a Low-FODMAP Diet on the Severity of Gastrointestinal Symptoms and Quality of Life in the Treatment of Gastrointestinal Disorders—A Systematic Review of Randomized Controlled Trials

**DOI:** 10.3390/nu17122045

**Published:** 2025-06-19

**Authors:** Laura Kuźmin, Katarzyna Kubiak, Ewa Lange

**Affiliations:** 1 Department of Dietetics, Institute of Human Nutrition Sciences, Warsaw University of Life Sciences (WULS-SGGW), Nowoursynowska 159c, 02-776 Warsaw, Poland; ewa_lange@sggw.edu.pl; 2 Department of Human Nutrition, Institute of Human Nutrition Sciences, Warsaw University of Life Sciences (WULS-SGGW), Nowoursynowska 159c, 02-776 Warsaw, Poland; katarzyna_kubiak@sggw.edu.pl

**Keywords:** FODMAPs, nutrition therapy, diet, treatment, gastrointestinal symptoms, quality of life, gastrointestinal disorders, gastrointestinal disease

## Abstract

**Background:** A low-FODMAP diet is considered as a potential supportive treatment approach in some gastrointestinal disorders. The aim of this study was to systematically review the literature for randomized controlled trials assessing the efficacy of the low-FODMAP diet on the severity of gastrointestinal symptoms and quality of life in patients with gastrointestinal disorders. **Methods:** This review was conducted in accordance with CASP tool and PRISMA guidelines. A comprehensive search of the PubMed, Scopus, and Web of Science databases resulted in the identification of fourteen randomized controlled trials. **Results:** Ten studies examined the effect of the low-FODMAP diet in patients with irritable bowel syndrome (IBS), three with inflammatory bowel disease (IBD), and one with symptomatic proton pump inhibitor (PPI) refractory gastroesophageal reflux disease (GERD). All interventions compared the low-FODMAP diet with another diet and lasted from 3 to 12 weeks. Most studies on IBS showed significant improvements in abdominal pain, bloating, and quality of life compared to control diets. In IBD, improvements were mainly observed in functional gastrointestinal symptoms, while no clear benefit was demonstrated in GERD. Heterogeneity in study designs, intervention durations, comparator diets, and outcome measures limited the ability to conduct a meta-analysis. **Conclusions:** Although a low-FODMAP diet may reduce symptoms in selected individuals, it is not universally necessary. Importantly, the diet’s restrictive nature and potential long-term effects—such as nutritional deficiencies and alterations in gut microbiota—highlight the need for clinical supervision by dietitians with expertise in gastrointestinal disorders. Furthermore, in some cases, symptom improvement may be achievable through less restrictive changes, such as improving food hygiene and reducing intake of processed or high-sugar foods. Further high-quality randomized controlled trials with standardized endpoints and longer follow-up are needed to clarify the efficacy and safety of the low-FODMAP diet across various gastrointestinal conditions.

## 1. Introduction

Gastrointestinal (GI) disorders, including irritable bowel syndrome (IBS) and inflammatory bowel diseases (IBD), affect a substantial proportion of the global population and are associated with a considerable burden on healthcare systems. These conditions are often characterized by chronic and recurring symptoms such as abdominal pain, bloating, diarrhea, and constipation, which significantly impair patients’ quality of life. GI disorders are multifactorial in origin, involving interactions between diet, the gut–brain axis, intestinal permeability, immune activation, and alterations in the gut microbiota [1]. While pharmacological treatments are available—such as antispasmodics, laxatives, anti-diarrheal agents, and biologics—their effectiveness varies, and some may be associated with adverse effects or limited long-term efficacy. In recent years, accumulating evidence indicates that alterations in the composition and metabolic activity of the gut microbiota play a pivotal role in the pathophysiology and symptom manifestation of GI disorders. The gut microbiota constitutes a complex and dynamic microbial ecosystem that resides within the gastrointestinal tract, exerting critical effects on host immune modulation, maintenance of intestinal barrier integrity, and fermentation of dietary components [1,2]. As a result, interventions targeting the microbiota, including dietary changes, have gained increasing interest as a promising therapeutic strategy for GI disorders.

One dietary strategy that has gained considerable attention in recent years is the low fermentable oligosaccharides, disaccharides, monosaccharides, and polyols (FODMAP) diet (LFD). This diet involves restricting the intake of specific short-chain carbohydrates that are poorly absorbed in the small intestine and rapidly fermented by gut bacteria, leading to gas production (hydrogen, methane), increased intestinal water content, and subsequent symptom exacerbation in susceptible individuals [2]. Several clinical studies suggest that a low-FODMAP diet can lead to significant symptom relief, particularly in patients with IBS, by reducing bloating, abdominal pain, and irregular bowel movements [3,4,5]. However, the diet’s long-term sustainability, nutritional adequacy, and effects on the gut microbiome remain subjects of ongoing debate [6].

Despite the growing body of literature on the low-FODMAP diet, the evidence regarding its overall efficacy remains inconsistent. While some randomized controlled trials (RCTs) report substantial improvements in symptom severity and quality of life, others indicate more modest benefits or highlight potential drawbacks, such as reduced microbial diversity in the gut [7,8,9,10]. The microbial dysbiosis induced by adherence to the LFD may have broader physiological implications beyond symptomatic improvement, as perturbations in gut microbiota composition have been associated with alterations in host immune responses, metabolic regulation, and neuropsychological health outcomes [3]. Moreover, variations in study design, patient populations, and outcome measures make it challenging to draw definitive conclusions about the diet’s clinical utility. While some guidelines recommend the LFD for IBS symptom relief, its role in other GI disorders, such as IBD or SIBO (small intestinal bacterial overgrowth), remains unclear and underexplored [4]. Previous reviews have either focused narrowly on IBS or included non-randomized or observational studies, limiting their ability to provide high-level evidence. To our knowledge, no systematic review of RCTs of the efficacy of LFD in GI disorders has been published without focusing exclusively on a single selected condition. Consequently, there is a need for an up-to-date synthesis focused exclusively on RCTs assessing the impact of the LFD across various GI disorders, especially since the LFD is now often recommended to patients by doctors and dietitians.

This systematic review aims to assess the efficacy of the low-FODMAP diet in alleviating GI symptoms and improving health-related quality of life in patients with GI disorders, based on evidence from randomized controlled trials.

## 2. Materials and Methods

This systematic review was registered at the protocol stage in the International Prospective Register of Systematic Reviews PROSPERO, Centre for Reviews and Dissemination, University of York, York, UK, record number CRD420251027143.

### 2.1. Search Strategy

A systematic search of the literature was conducted in agreement with the Preferred Reporting Items for Systematic reviews and Meta-analysis (PRISMA 2020) statement [11]. Two investigators independently (L.K. and K.K.) searched PubMed, Scopus, and Web of Science for relevant studies up to April 2025 using variations and combinations of the following search terms: low-FODMAP diet, fermentable oligosaccharides, disaccharides, monosaccharides and polyols, gastrointestinal disorders, gastrointestinal symptoms, diet, treatment, effect. The search strategy is shown in Table S1.

### 2.2. Inclusion and Exclusion Criteria

The studies had to comply with the following inclusion criteria: randomized controlled trial; adult population (≥18 years old); comparison of the use of the low-FODMAP diet with any other diet/nutrition method; studies reported in English; studies from the last 10 years.

The exclusion criteria were as follows: children and adolescents (<18 years old); pregnant or breastfeeding women; athletes (people training professionally); enteral nutrition; no information about what the LFD looked like; comparison of the use of LFD with a diet enriched with a supplement, probiotic, etc.; using another elimination diet at least 3 months before starting LFD; using LFD and another intervention simultaneously; RCTs with crossover design; case studies/reports; conference abstracts; letters.

### 2.3. Study Selection and Data Extraction

L.K. and K.K. independently screened the obtained records by title and abstract after removing duplicates, and then a full-text evaluation was based on the predefined criteria. Inconsistencies were resolved by discussion between the two investigators.

Extracted data included the following: name of the author(s), year of publication, study location, study design, number of participants in an intervention and control group, and inclusion and exclusion criteria for both groups. Furthermore, the information necessary in terms of results included characteristics of the diet used in both groups, period of intervention, type of disease/illness, results of the impact of the intervention on selected parameters before and after, and *p*-values within groups before and after the intervention as well as between the intervention and control groups. If the necessary data were missing in the articles, the authors were contacted to obtain them.

Due to substantial heterogeneity across the included studies in terms of study populations, intervention protocols, comparator diets, outcome measures, and follow-up durations, a quantitative meta-analysis was deemed inappropriate. Instead, a narrative synthesis was conducted in accordance with the Critical Appraisal Skills Programme (CASP UK, Oxford, UK; 2020 version) Randomised Controlled Trial Standard Checklist [12] and PRISMA guidelines [11], summarizing and interpreting the findings qualitatively.

### 2.4. Quality Assessment

Randomized studies were assessed using the CASP Randomised Controlled Trial Standard Checklist [12]. Due to the specificity of research conducted with the participation of a dietitian and patients, which requires the introduction of specific dietary recommendations, we decided that in order to define a study as having minor limitations, it would be sufficient to blind only one of the three groups of people involved in the study (participants, investigators, or people assessing/analyzing outcomes). An overall assessment of the study limitations was made as follows: when at least one of the groups (participants or investigators or people assessing/analyzing outcomes) was blinded and the assessments for the rest of the items in the tool were not “no”, these were considered minor limitations; when the assessments for one or more questions in the tool (except questions about blinding) were “no”, these were considered as major limitations.

All studies included in this review reported approval by an appropriate institutional ethics committee, thereby adhering to established ethical standards for research involving human subjects.

## 3. Results

### 3.1. Trial Selection

**Figure 1:**
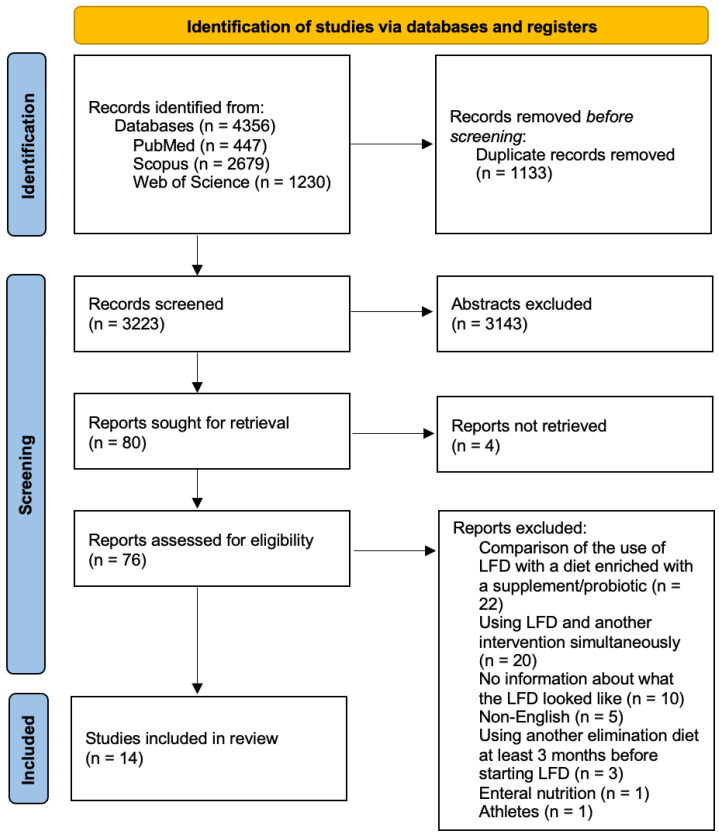
Literature review flow diagram of the selection publication process according to the Preferred Reporting Items for Systematic Reviews and Meta-Analyses (PRISMA) statement [11].

In total, 4356 articles were found and 1133 were duplicates. After screening for abstracts, 3143 articles were excluded. A total of 14 studies were eligible for inclusion. A PRISMA flow diagram shown in Figure 1 presents the results of the search.

### 3.2. Characteristics of the Trials

Table 1 provides the characteristics of the trials included in this systematic review. All the papers were published after 2015. Eight of them were published in the last 5 years [7,13,14,15,16,17,18,19]. Eight RCTs were conducted in Europe (two each in Italy [17,20] and the UK [13,15], one each in Sweden [21], Denmark [22], France [16], and Poland [14]), five in Asia (two in China [7,19], one each in Iran [23], Turkey [18], and Thailand [24]), and one in the US [25]. The total number of included participants varied from 31 in the Rivière trial [16] to 121 recruited by Tunali [18]. The intervention group with the smallest number of participants comprised 16 individuals [16], and with the largest 54 people [7]. Ten studies included patients with IBS [7,14,15,17,18,19,21,23,24,25] (including four studies with patients with IBS-D (irritable bowel syndrome with diarrhea) [7,17,23,25], one with patients with IBS-M (irritable bowel syndrome mixed type) [14], one with patients with IBS-D or IBS-M [15], and four with patients with IBS without division into disease type [18,19,21,24]), three studies included patients with IBD [13,20,22] (one study with patients with IBD in remission or with mild disease activity [20], one study with patients with quiescent IBD [13], and one study with patients with IBD in remission or with mild-to-moderate disease and coexisting IBS-like symptoms [22]), and one study with patients with symptomatic PPI (proton pump inhibitor) refractory GERD (gastroesophageal reflux disease) [16]. In most studies, the LFD was followed for 4 [13,15,16,19,21,24,25] or 6 weeks [18,20,22,23]. The longest duration of the LFD was 12 weeks [17], while the shortest duration was 3 weeks [7]. In three studies, people in the control group followed a diet consistent with recommendations for people with IBS [21,24,25]. In eight studies, people in the control group were recommended a standard, normal diet, with traditional dietary advice [7,13,15,16,19,20,22,23], including one study in which a second control group followed a GFD (gluten-free diet) [15]. Three studies used different dietary approaches. In one of them, the control group consisted of people on a diet with food based on Tritordeum flour [17]. In the second study, two control groups were included: one used the IgG (immunoglobulin G)-based elimination–rotation diet and the other used an easy digestible diet [14]. In the third study, the control group followed a diet designed based on AI-recommended foods derived from microbiome analysis results [18].

### 3.3. Study Quality

The specifics of the quality evaluation for the randomized studies are presented in Table S2. Eight of randomized controlled trials [13,14,15,16,21,22,23,25] were determined to exhibit major limitations, while six studies [7,17,18,19,20,24] were identified as having minor limitations. According to the CASP tool [12], the primary issues identified in studies demonstrating major limitations were as follows: no blinding of people participating in the study, not all participants who entered the study were accounted for at its conclusion, not all studied groups received the same level of care, and the effects of an intervention were not comprehensively reported.

### 3.4. Outcomes of Interest

The severity of symptoms was assessing by the IBS Symptom Severity Scale (IBS-SSS) [7,13,15,16,17,18,19,21,22,23,24] and the frequency by interviewing patients using closed questions with a single choice: “yes” or “no” [14]. Cox et al. [13] also used the Gastrointestinal Symptom Rating Scale (GSRS) to assess gut symptoms. The severity of upper and lower gastrointestinal symptoms in Patcharatrakul et al. [24] was assessed using a 0–10 cm visual analog scale (VAS).

Changes in the quality of life of participants were assessed using IBS-specific tools like the IBS-associated quality of life (IBS-QOL) [7,14,18,23,25], Inflammatory Bowel Disease Questionnaire (IBD-Q) [20], UK-specific Inflammatory Bowel Disease Questionnaire (UK IBD-Q) [13], Short Inflammatory Bowel Disease Questionnaire (SIBDQ) [22], and GIQLI (Gastrointestinal Quality of Life Index) [16].

The level of anxiety or depression was measured using the Hospital Anxiety and Depression Scale (HADS) [15,16,18,25]. In one study [7], the severity of anxiety was measured by General Anxiety Disorder (GAD-7).

Eswaran et al. [25] also assessed work productivity using a Work Productivity and Activity Impairment questionnaire and sleep quality using a numerical rating scale for sleep quality and fatigue.

The Patient Health Questionnaire (PHQ-12) non-GI somatic symptoms scale was measured in two studies [7,15].

IBD activity was defined using the partial Mayo score [13,20] and the Harvey–Bradshaw index (HBi) [13,20,22] for patients with UC and CD, respectively, while Pedersen et al. [22] measured the UC activity by the Simple Clinical Colitis Index (SCCAI). Modifications in subclinical intestinal inflammation were assessed by fecal calprotectin values and C-reactive protein (CRP) levels [13,20,22].

In order to diagnose GERD, Riviere et al. [16] used the Reflux Disease Questionnaire (RDQ).

### 3.5. Effect of the LFD in IBS

The influence of the LFD in IBS was assessed in ten studies [7,14,15,17,18,19,21,23,24,25] (Table 2). Five of them [14,15,21,23,25] were considered to have major limitations; the remaining five studies [7,17,18,19,24] were assessed as having minor limitations.

Böhn et al. [21] observed that the IBS-SSS scores decreased in both groups by the end of the intervention compared to the baseline. A reduction in the severity of IBS symptoms was already observed at day 14 in both groups. The decrease was statistically significant in the LFD group (*p* = 0.002), while the traditional IBS diet group showed a trend toward improvement (*p* = 0.051). The proportion of responders, defined as a reduction in IBS-SSS ≥50, was similar between the treatment groups, with 19 patients (50%) in the LFD group and 17 patients (46%) in the traditional IBS diet group. When examining the impact of the interventions on the individual IBS-SSS items, all aspects showed improvement in both groups by day 29 compared to the baseline. Statistically significant improvements were observed in both groups for abdominal pain frequency (respectively, for the LFD group: *p* = 0.008; for the control group: *p* < 0.001), abdominal distention severity (LFD group: *p* < 0.001; control group: *p* = 0.003), and life interference (LFD group: *p* = 0.001; control group: *p* = 0.002). Additionally, bowel habit dissatisfaction significantly improved in the control group (*p* = 0.01). In the LFD group, there was a significant reduction in the number of daily bowel movements by the end of the treatment (*p* < 0.001). However, there were no significant differences between the two groups.

Eswaran et al. [25] demonstrated that the average IBS-QOL score after the intervention showed a significant improvement in both groups; however, the improvement was notably greater in the LFD group compared to the control group. Significant improvements were seen across all IBS-QOL domains in the LFD group (interference with activity, dysphoria, body image, social reaction, health worry, sexual, relationship) except for food avoidance (*p* < 0.05). The mNICE (modified diet recommended by the National Institute for Health and Care Excellence) group also exhibited improvements in several domains, including dysphoria, interference with activity, and health worry. The LFD group achieved a meaningful clinical response (MCR) in the domains of interference with activity, dysphoria, body image, and social reaction, whereas the mNICE group did not attain MCR in any IBS-QOL domain. After the intervention, the proportion of patients with MCR (at least a 14-point improvement) was greater in the case group than in the control group (52% vs. 21%; 95% CI, −0.52 to −0.08). After 4 weeks, anxiety and depression scores improved significantly only in the LFD group compared with the baseline. The difference between groups in terms of improvement in results was statistically significant only for anxiety scores (1.63; 95% CI, 0.46–2.80). The proportion of patients with anxiety scores <8 did not differ significantly between the LFD group (52%; 95% CI, 0.37–0.68) and the mNICE group (37%; 95% CI, 0.21–0.53). The proportion of patients with depression scores <8 was comparable between the low-FODMAP group (85%; 95% CI, 0.67–0.93) and the mNICE group (80%; 95% CI, 0.73–0.96). Analyzing the Work Productivity and Activity Impairment scores, only the activity impairment scores showed improvement with both diet interventions at 4 weeks compared to the baseline. However, the LFD group reported a significantly greater benefit compared to the control group (13.50; 95% CI 2.72, 24.20). Absenteeism, presenteeism, and overall work impairment measured at the baseline and post-intervention did not show significant changes following the low-FODMAP diet or mNICE. When it comes to sleep- and fatigue-related variables, in comparison to the baseline, after 4 weeks the LFD group showed significant improvements in mean sleep and fatigue scores, although the difference in the level of change between the two groups was not statistically significant (95% CI, −0.29 to 1.20; 95% CI, −0.46 to 1.13, respectively). According to the modified sleep questionnaire, the LFD group showed improvements in overall sleep quality, including daytime fatigue (95% CI, −0.56 to −0.07) and difficulty falling asleep (95% CI, −0.65 to −0.03), at 4 weeks compared to the baseline. However, the difference between the two groups was not statistically significant (95% CI, −0.17 to 1.58; 95% CI, −0.09 to 0.61; 95% CI, −0.09 to 0.67).

Liu et al. [19] noticed that following the intervention, the LFD group exhibited significant relief in the IBS-SSS at both 2 weeks (37.5%) and 4 weeks (44.2%) compared to the baseline (*p* < 0.05). At 4 weeks, the IBS-SSS was significantly improved in the LFD group compared to the control group. Among the symptoms related to the IBS-SSS, abdominal pain, pain frequency, bloating, and the satisfaction of the bowel habits were significantly improved in the LFD group at both the 2 weeks and 4 weeks compared with the baseline (*p* < 0.05) (respectively, baseline vs. after 4 weeks: 26.39 ± 21.82 vs. 9.72 ± 15.19; 28.33 ± 33.08 vs. 8.33 ± 13.39; 31.67 ± 24.07 vs. 16.67 ± 14.85; 63.06 ± 22.71 vs. 48.11 ± 17.38). At 4 weeks, the pain frequency was significantly improved in the LFD group compared with the control group (*p* < 0.05). The remaining symptoms showed no significant difference between groups. After 2 weeks and 4 weeks of intervention, the life impact was significantly improved in the LFD group as compared to the baseline (*p* < 0.05), but there was no significant difference among the groups at 2 weeks and 4 weeks.

Ostrowska et al. [14] assessed the presence of “typical” clinical symptoms, dyspeptic symptoms, and extraintestinal symptoms, and patients were asked closed questions with one choice: “yes” or “no”. Statistically significant differences were observed in the G2-IP group (group 2 immunoglobulin G (IgG)-based elimination–rotation diet) but not in the G1-FM group (group 1 low-FODMAP diet) or control group when comparing idiopathic abdominal pain, abdominal pain after a meal, abdominal pain during defecation, and the sensation of incomplete defecation before and after the interventions. Analyzing the presence of mucus in the stool, in the final examination, a significant improvement was observed only in the G1-FM group (respectively, number of patients before vs. after the intervention: *n* = 8 vs. *n* = 2, *p* = 0.031) and in the G2-IP group, where the symptom was completely absent (*n* = 6 vs. *n* = 0). However, the percentage of patients reporting mucus in the stool increased in the control group, although this change was not statistically significant (*n* = 5 vs. *n* = 6). The number of patients reporting blood in the stool decreased in groups G1-FM (*n* = 3 vs. *n* = 0) and G2-IP (*n* = 2 vs. *n* = 0) but did not change in the control group (*n* = 2 vs. *n* = 2). After the intervention, significant improvement in difficulty to defecate (constipation) was present only in the G2-IP group. On the other hand, bloating, gurgling sensation, and gastric fullness decreased significantly in the G1-FM and G2-IP groups. Dyspeptic IBS symptoms, such as heartburn and belching, improved significantly only in the G2-IP group (respectively, *n* = 7 vs. *n* = 1, *p* < 0.03 and *n* = 6 vs. *n* = 0). At the final assessment, nausea resolved in the G1-FM (*n* = 6 vs. *n* = 0) and G2-IP (*n* = 7 vs. *n* = 0) groups but persisted in the control group (*n* = 9 vs. *n* = 9). The number of participants experiencing constant tiredness and weakness was significantly reduced only in the G2-IP group (*n* = 7 vs. *n* = 1, *p* = 0.031). The skin condition improved and headaches/migraines occurred less frequently in the G2-IP group (respectively: *n* = 4 vs. *n* = 0; *n* = 3 vs. *n* = 0), while in the G1-FM group and the control group, the number of participants did not change or decreased insignificantly (respectively: *n* = 0 vs. *n* = 0; *n* = 3 vs. *n* = 3; *n* = 1 vs. *n* = 1; *n* = 5 vs. *n* = 4).

Patcharatrakul et al. [24] in their study defined responders as those who achieved at least a 30% reduction in the average level of their worst daily abdominal pain or discomfort after a period of 4 weeks. Following SILFD (structural individual low-FODMAP dietary advice), 60% of patients (18 out of 30) met the responder criteria compared to 28% (9 out of 32) after BRD (brief advice on a commonly recommended diet) (*p* = 0.001). Upper and lower gastrointestinal symptom severity, including abdominal pain, abdominal discomfort, belching, bloating, and stool urgency, was measured using a 0–10 cm visual analog scale (VAS) at the baseline and at the end of the intervention. A significant reduction in the IBS-SSS was observed only following the SILFD diet compared to the baseline values. Post-intervention, the IBS-SSS in the SILFD group was significantly reduced compared to the BRD group. Significant decreases in the severity of abdominal pain, abdominal discomfort, and bloating were observed compared to the baseline solely after SILFD (respectively: 0.001; <0.001; 0.02). Nevertheless, after the interventions, these symptoms did not differ significantly between the case and control groups. Belching and stool urgency following SILFD and BRD interventions remained unchanged compared to the baseline. Of the 33 patients with IBS-C, 15 were assigned to the SILFD group and 18 to the BRD group. A response was observed in 73% (11/15) of patients following SILFD, compared to 28% (5/18) in the BRD group (*p* < 0.05). Global IBS symptoms, abdominal pain, and abdominal discomfort severity scores after SILFD were significantly lower than those in the BRD group (global symptom severity, SILFD vs. BRD: (VAS 0–100) 35.1 ± 19.6 vs. 53.0 ± 21.7, *p* < 0.05; abdominal pain, SILFD vs. BRD: (VAS 0–10) 1.2 (0–3) vs. 4.1 (0–5.7), *p* < 0.05; abdominal discomfort, SILFD vs. BRD: 2.5 (0.8–4.8) vs. 5 (2.6–6.7), *p* < 0.05). Post-intervention bloating and belching did not differ significantly between the SILFD and BRD groups. Similarly, stool frequency in the fourth week after the interventions showed no significant difference between the two groups. Among 29 patients with IBS-non-C, 15 were assigned to SILFD and 14 to BRD. A response was observed in 47% (7/15) of SILFD patients and 29% (4/14) of BRD patients (*p* < 0.05). Post-intervention, there were no significant differences between groups in global IBS symptom severity, abdominal pain, discomfort, bloating, belching, or stool urgency or frequency.

Rej et al. [15] observed that a ≥50-point reduction in the IBS-SSS, the primary endpoint, was achieved by 42% (14/33) of TDA (traditional dietary advice) patients, 55% (18/33) of LFD patients, and 58% (19/33) of GFD patients, with no significant differences among the groups. Among patients achieving a ≥50-point reduction in the IBS-SSS, significant within-group improvements were observed in individual IBS-SSS components across all dietary interventions, with no significant differences between groups. A ≥50-point reduction in the IBS-SSS was observed in 54% (40/74) of IBS-D patients and 44% (11/25) of IBS-M patients, with no significant difference between groups. Response rates did not differ between IBS-D and IBS-M based on dietary therapy. Interestingly, a reduction of ≥50 points in the IBS-SSS occurred in 52% (*n* = 15/29) of individuals receiving face-to-face consultations compared to 51% (*n* = 36/70) in the live virtual consultation group (*p* = 0.98). This effect was observed to a similar degree regardless of the assigned dietary therapy. Individuals assigned to the LFD experienced a significant improvement in depression compared to the TDA and GFD groups (*p* = 0.03). Abdominal pain severity, numbers of days in pain every 10 days, abdominal distention severity, satisfaction with bowel habits, and interference with life in general decreased after the intervention, but no significant difference was observed between groups. In the IBS-QOL questionnaire analysis, a significant difference was noted within the LFD group, where participants reported significant improvement only in dysphoria when compared to other groups (*p* < 0.01). No significant differences were observed across groups in changes in anxiety or somatization. Participants assigned to the LFD showed a significant reduction in depression symptoms compared to those in the TDA group.

Russo et al. [17], comparing the total IBS-SSS score, found statistically significant differences in both groups before and after the intervention. The IBS-SSS total score significantly decreased after four weeks of treatment, with a further, though less pronounced, reduction in the subsequent weeks. No differences were observed between the groups. By the end of the intervention, all individual IBS-SSS items showed improvement in both groups compared to the baseline measurements. A time effect was observed, with no differences between groups. After 12 weeks of intervention, the following changes occurred in the LFD and TBD groups: severity of abdominal pain (reduced by −25.8; *p* < 0.0001 and −24.2; *p* < 0.0001, respectively), frequency of abdominal pain (reduced by −26.7; *p* < 0.0001 and −23.7; *p* = 0.0007), severity of abdominal distension (reduced by −28.1; *p* < 0.0001 and −30.9; *p* < 0.0001), dissatisfaction with bowel habit (reduced by −27.9; *p* = 0.0006 and −28.2; *p* = 0.0005), and interference with quality of life (reduced by −23.7; *p* = 0.0012 and −23.4; *p* = 0.0015). The study also analyzed anthropometric, bioelectrical impedance (BIA), and biochemical measurements before and after the 12-week period of the two diets. Following the intervention, a time effect was observed, with no differences between the two diets regarding anthropometric and BIA parameters. Significant reductions were noted in various anthropometric measures, including weight, body mass index (BMI), abdominal and waist circumferences, as well as BIA parameters such as fat mass (FM), free fat mass (FFM), total body water (TBW), and extracellular water (ECW). Over time, both fasting glucose and C-reactive protein (CRP) levels significantly decreased (*p* < 0.05), with no significant differences observed between the two diet groups. Conversely, vitamin D levels increased over time (*p* = 0.04), with no variation between groups in terms of administration timing. None of the diets influenced the changes in the lipid profiles and micronutrient profile.

Tunali et al. [18] noticed that the IBS-SSS score was significantly lower in both groups after the intervention compared to the baseline but with no difference between the groups. Significant improvements were observed in both the case and control groups in abdominal pain severity (*p* < 0.001), abdominal pain frequency (*p* < 0.001), abdominal distension severity (*p* < 0.001), and bowel habits dissatisfaction (*p* < 0.001). Life interference in general, caused by IBS symptoms, significantly decreased in the control (*p* < 0.001) and case groups (*p* = 0.02). However, the differences between groups were not significant in any of the above. Daily stool frequency did not change significantly in either group when comparing the baseline and end of the study. Significant changes were also noted in quality of life and levels of depression and anxiety but with no differences between groups. By analyzing each of the IBS subtypes, IBS-SSS scores significantly improved from the baseline to 6 weeks across all subtypes and dietary interventions. In IBS-C and IBS-M, both diets led to a marked reduction in IBS-SSS scores (*p* < 0.001). For IBS-D, the PD diet resulted in a more substantial decrease (*p* = 0.010) compared to the LFD (*p* = 0.312). At 6 weeks, the PD significantly improved IBS-QOL scores for IBS-C (*p* < 0.001), IBS-D (*p* < 0.001), and IBS-M (*p* = 0.008). In contrast, LFD led to significant improvements in IBS-C (*p* = 0.004) and IBS-D (*p* = 0.022), but not in IBS-M (*p* = 0.646), compared to the baseline.

Zahedi et al. [23] observed that IBS-SSS scores were significantly lower in both groups, but the difference was significantly greater in the LFD group. At the end of the study, both groups exhibited a significant reduction in abdominal pain intensity and frequency, abdominal distension, dissatisfaction with intestinal transit, interference with daily life, and bowel habit parameters (stool frequency and consistency) compared to the baseline (*p* < 0.001 in both groups). The individual IBS-SSS items showed significant reductions in the LFD group compared to the GDA (general dietary advice) group, with abdominal pain intensity (*p* = 0.001), abdominal pain frequency (*p* = 0.017), abdominal distension (*p* < 0.001), dissatisfaction with intestinal transit (*p* = 0.001), and interference with daily life (*p* = 0.005) all significantly improved. Additionally, bowel habit indicators, including stool consistency (*p* = 0.003) and stool frequency (*p* < 0.001), significantly decreased in the low-FODMAP group compared to the control group. The IBS-QOL scores decreased in both groups by the end of the intervention compared to the baseline, with no differences between the groups.

Zhang et al. [7] defined primary endpoint as a ≥50-point reduction in the IBS-SSS. They showed it was met in 59% (30/51) of patients in the LFD group compared with 53% (26/49) in the TDA group. In the case group, after the week, significant improvements were observed in abdominal pain (*p* = 0.007), stool frequency (*p* = 0.002), excessive wind (*p* < 0.001), and urgency (*p* = 0.003). In the same group, after 3 weeks, symptoms like abdominal pain (0.009), stool frequency (*p* < 0.001), excessive wind (*p* < 0.001), urgency (*p* = 0.003), and incomplete defecation (*p* = 0.001) demonstrated significant improvement. In contrast, in the control group, the improvements after 3 weeks were observed in abdominal pain (*p* = 0.002), stool consistency (*p* = 0.021), excessive wind (*p* = 0.021), and urgency (*p* = 0.028). However, no significant differences in individual symptoms between the two groups were observed in the third week. Both dietary interventions were well adhered to and resulted in significant improvements in quality of life and mental health, with no differences between the groups.

### 3.6. Effect of the LFD in IBD

The influence of the LFD in IBD was assessed in three studies [13,20,22] (Table 3). Two of them [13,22] were considered to have major limitations; only one study [20] was assessed as having minor limitations.

Bodini et al. [20] reported a significant reduction in the median HBi in the LFD group after the intervention, whereas no such change was observed in the SD (standard diet) group. Additionally, no statistically significant difference in HBi between the groups was identified. Following the 6-week dietary intervention, the scores showed a numerical but non-significant decrease in the LFD group while remaining largely unchanged in the SD group. No significant difference between the groups was observed at this time point. After dietary intervention, a statistically significant reduction in median calprotectin values was observed in the LFD group, whereas no significant change was detected in the SD group. However, no statistically significant difference between the groups was observed. Overall, between the beginning and end of the intervention, the median calprotectin values decreased by 34.7% in the LFD group and by 4.4% in the SD group. Median CRP values remained similar before and after the 6-week dietary intervention in both the LFD group (post-intervention: 3.1 mg/L; IQR, 2.5–7.6 mg/L) and the SD group (post-intervention: 3.1 mg/L; IQR, 1.7–4.2 mg/L). No significant difference between the groups was observed at the end of the intervention. Following the 6-week dietary intervention, a modest yet statistically significant increase in median IBD-Q was observed in the LFD group, whereas no significant change was detected in the SD. However, the difference between groups at the end of the intervention was not statistically significant. Additionally, the proportion of patients with an IBD-Q >170 increased in the LFD group from 42.3% (11/26) at the baseline to 50% (13/26) post-intervention, while it remained unchanged in the SD group (65%, 19 of 29 patients at both time points). No significant changes were detected in the various IBD-Q subcategories in either study group.

Cox et al. [13] noted a greater reduction in the total IBS-SSS score following the LFD compared to the SD; however, this difference was not statistically significant. The analysis of IBS-SSS subscores showed that participants following the LFD had a significantly lower score only for bloating severity than participants on an SD (respectively: 23, estimated marginal mean [SEM] 3; 34, SEM 3; *p* = 0.021). However, the exploratory analysis showed that a significantly higher number of participants achieved a 50% reduction in the IBS-SSS after following the LFD (9/27, 33%) compared to the SD (1/25, 4%; *p* = 0.012). All clinical outcomes were also assessed through predefined subgroup analyses for UC (*n* = 26) and CD (*n* = 26). Considering only UC patients, after the intervention, the total IBS-SSS score was significantly lower in the LFD group than in the SD group (respectively: 135, SEM 15; 183, SEM 15; *p* = 0.031). No significant difference was observed among CD patients. There were also no significant changes in the IBS-SSS subcategories when analyzing only patients with UC or only patients with CD. A significantly greater proportion of patients reported adequate relief of gut symptoms following the LFD (14/27, 52%) compared to the SD (4/25, 16%; *p* = 0.007). No differences were observed in the proportion of patients reporting adequate relief between the LFD and SD in the subgroup analyses of UC or CD. Using the GSRS, no differences in the incidence or severity of any symptoms were observed between the groups except for the severity of flatulence, which was significantly lower after following the LFD (0.9, SEM 0.1) than the sham diet (1.2, SEM 0.1; *p* = 0.035). A significant reduction in daily stool frequency was observed following the LFD compared to the SD. However, there was no significant difference in the proportion of stools with normal consistency between the low-FODMAP diet (65%, SEM 5%) and the sham diet (69%, SEM 5%). The total UK IBDQ score, reflecting an improved HR-QOL, was significantly higher after the LFD compared to the sham diet. In particular, the Bowel II domain score, which measures the impact of GI symptoms on HR-QOL, was also significantly greater following the LFD (76.5, SEM 2.0) compared to the sham diet (70.0, SEM 2.1, *p* = 0.031). In both CD and UC, no difference was observed in the Harvey–Bradshaw index score and partial Mayo score, respectively, between the low-FODMAP and sham diet at the end of the trial. No difference was observed in end-of-trial fecal calprotectin levels between the LFD and SD nor in serum CRP concentrations between the LFD (2.0 mg/L, SEM 0.3) and the SD (1.6 mg/L, SEM 0.3). The IBD-control score showed a higher patient-perceived control of IBD following the LFD (88.3, SEM 4.3) compared to the SD (74.3, SEM 4.5; *p* = 0.028). This difference was particularly evident in UC (94.2, SEM 6.6 vs. 71.3, SEM 6.6; *p* = 0.022) but not in CD (81.4, SEM 5.2 vs. 79.1, SEM 5.7).

Pedersen et al. [22] noticed that 30 patients (81%) in the LFD group demonstrated a response to treatment compared to 19 patients (46%) in the ND group (*p* < 0.01). The total IBS-SSS score was significantly lower after the intervention compared to the baseline in both groups. Moreover, there was a significant difference between the LFD and ND group. An analysis of IBS-SSS subscores proved that patients from the LFD group had a significantly greater reduction in pain duration, along with a trend toward improved stool frequency and consistency, compared to those on an ND (respectively: OR = 2.97, 95% CI: 1.12–7.89, *p* = 0.03; OR = 2.43, 95% CI: 0.97–6.12, *p* = 0.06). According to disease activity, the subgroup analysis of the IBS-SSS showed that only IBD patients in remission had a significantly better response on the LFD (median IBS-SSS: 105, IQR 26–167) than on the control diet (median IBS-SSS 175, IQR 77–298), *p* < 0.01. No significant difference in the IBS-SSS was observed among IBD patients with mild-to-moderate disease activity between the LFD group (median IBS-SSS 169, IQR: 105–332) and the ND group (median IBS-SSS 140, IQR: 96–211). Moreover, only CD patients following LFD had a significantly lower total IBS-SSS score (median IBS-SSS 58, IQR 18–173) compared to the ND group (median IBS-SSS 220, IQR 57–357), *p* = 0.02, whereas in the UC group, no effect of diet was observed LFD (LFD: median IBS-SSS 120, IQR 36–170 vs. ND: median IBS-SSS 141, IQR 52–263). After 6 weeks, a significant difference in IBS-QOL was observed only in the LFD group, but no significant change was observed between the groups. SIBDQ scores showed a statistically significant improvement in the LFD group but not in the ND group (compared to the baseline), with a significant difference between groups at the end of the study. HBi and SCCAI decreased only in the case group after the intervention relative to the baseline. There were no significant differences observed between the case and control groups in HBi reduction. In contrast, patients following the LFD showed a significantly greater reduction in SCCAI scores compared to those on the ND. No significant difference in geometric mean FC (fecal calprotectin) was observed in the LFD nor in the ND group at week 6 compared to the baseline. Similarly, no significant difference in FC change between the LFD and ND groups was observed at the end of the study. A significant increase in geometric mean CRP was observed in the ND group at week 6 (2.6; 95% CI: 2.1–3.3) compared to the baseline (2.2; 95% CI: 1.8–2.5; *p* = 0.04). In contrast, no significant change in mean CRP was noted in the LFD group at week 6 compared to the baseline. Additionally, no significant difference in CRP changes between the LFD and ND groups was observed at the end of the study.

### 3.7. Effect of the LFD in Symptomatic PPI Refractory GERD

Only one study examined the impact of the LFD in symptomatic PPI refractory GERD [16] (Table 4). The quality of the study was assessed as having major limitations.

Riviere [16] showed no statistically significant difference in response rates between the LFD and UDA (usual dietary advice) groups. The median total score of the RDQ exhibited a statistically significant decrease over time within both cohorts, with no difference according to the assigned diet group. The median (IQR) IBS-SSS decreased significantly for the LFD group only, with no differences between the groups. No statistically significant variation in GIQLI scores was observed between the pre-diet and post-diet assessments in both groups. After the intervention, no significant differences were observed in pH-impedance parameters between the LFD and UDA groups. This included the total number of reflux events, total acid exposure, total bolus exposure, and the proportion of patients with a positive symptomatic association. At week 4, 12/16 patients (75%) in the LFD group and 12/15 patients (80%) in the UDA group continued to exhibit abnormal pH-impedance monitoring (*p* = 1.0). Among the 9/31 patients who achieved a clinical response, 4 of them exhibited negative pH-impedance monitoring at week 4, while 5 had positive pH-impedance results. Of the five patients who demonstrated both clinical response and normalization of pH-impedance monitoring, four belonged to the LFD group. According to the HADS assessment, 4 out of all 31 participants (13%) met the criteria for subclinical depression, while 10 out of all 31 (32%) exhibited symptoms indicative of subclinical anxiety.

## 4. Discussion

This systematic review of the literature assessing the impact of the low-FODMAP diet considers all digestive system diseases and conditions. In this review, the information collected focused on three disease entities: IBS [7,14,15,17,18,19,21,23,24,25], IBD [13,20,22], and GERD [16]. While the included RCTs differed in some respects, common patterns emerged across several studies.

In the case of IBS, it was noted that the low-FODMAP diet reduced the severity of gastrointestinal symptoms [14,17,18,19,21,23,24] (assessed based on the IBS-SSS results). The most common positive changes concerned symptoms such as severity and frequency of abdominal pain, and bloating. Those results are consistent with Black et al. (2022) [2], who noticed that in terms of its effects on individual symptoms, a low-FODMAP diet was superior to alternative dietary advice for abdominal pain severity, abdominal bloating, or distension severity. Moreover, Khan et al. [39] in a 2025 meta-analysis covering 36 studies also noted that most studies confirm significant improvement in symptoms in patients on a low-FODMAP diet compared to other diets. In another meta-analysis, Khalighi Sikaroudi et al. [3] noted that the LFD significantly clinically improved all symptoms according to the IBS-SSS questionnaire within 4 weeks except for stool consistency, which needed more than 4 weeks of LFD implementation. However, it is worth considering that in some studies analyzed in this review [14,17,18,21], improvement in symptom severity also occurred in control groups, and no statistically significant differences were observed between groups. Significant changes were observed in people using the following: diet with Tritordeum-based foods; personalized diet designed based on AI-recommended foods derived from microbiome analysis results; IgG-based elimination–rotation diet; and two diets based on dietary recommendations from the British Dietetic Association, in which special attention was paid to eating meals in peace and to chewing thoroughly. This may suggest that the improvement in symptoms was related to a general change in dietary habits and food hygiene, paying more attention to choosing less processed products and those that do not aggravate symptoms or personalizing the diet, and not necessarily to the elimination of FODMAP products.

In terms of quality of life in IBS patients, as measured by the IBS-QOL, the results were fairly consistent in this analysis. Three studies found significant improvements in quality of life in both the LFD and the control diet, with no difference between groups [7,18,23]. The control diets in these studies were based on personalized AI-recommended foods derived from microbiome analysis results and/or attention to calm eating and chewing thoroughly. As before, this may suggest that a personalized diet and/or one based on changes in food hygiene (like mindful eating and precise mechanical processing of food in the oral cavity) may be as effective as the LFD. Only in one study, where the reference diet was based on the mNICE recommendations, were there significant differences between groups in favor of the low-FODMAP diet [25]. Similar results were obtained by Harvie et al. [40], who also observed higher mean IBS-QoL in the LFD compared to the control, but in this case the control group did not receive any nutritional education. The meta-analysis by van Lanen et al. [1] found a statistically significant five-point improvement in quality of life when comparing subjects on an LFD to those on a control diet. Whether this reflects a meaningful change in health-related QoL is unclear, as a 10-point change was previously considered clinically relevant [41]. Several studies assessed the effects of a low-FODMAP diet on anxiety and depression, but the results were inconsistent [7,15,18,25]; however, in most cases, improvement was seen with the use of an LFD, with no difference between groups. The discrepancy in results may have been influenced by the use of different questionnaires to measure anxiety and depression levels. Different results were obtained in a meta-analysis of over 1600 people with IBS, where the LFD was found to significantly improve quality of life but not in the anxiety and depression state [3]. It is worth adding that in our review, one of the studies also assessed the effect of the LFD on sleep-related parameters, where a significant difference in improvement was observed, as well as on work-related parameters, the where LFD had no effect on their improvement [25].

In recent years, the use of a low-FODMAP diet has expanded to patients with IBD, who continue to experience similar gastrointestinal symptoms despite clinical disease remission. In the case of IBD, the results regarding the reduction of gastrointestinal symptoms with LFD were not consistent in any of the studies analyzed, although a downward trend in IBS-SSS scores was observed in both groups [13,22]. Peng et al. [42] also found that LFD improved overall functional gastrointestinal symptoms. It also resulted in higher quality-of-life scores and lower Crohn’s disease HBi scores. However, there were no statistically significant differences in normal stool consistency, Mayo ulcerative colitis score, or fecal calprotectin. In our analysis, quality-of-life scores indicated improvement with LFD, but there was not always a significant difference between groups [13,20,22]. Similar results were obtained by Lamb et al. [43], who observed a significant decrease in IBS-SSS scores and a significant increase in SIBDQ and IBD-Q scores with the low-FODMAP diet; however, there were no significant changes in the control group after treatment.

In our analysis, a significantly lower HBi score, but not Mayo score, was also observed after the LFD, suggesting that this dietary intervention may be beneficial in reducing disease activity in patients with Crohn’s disease but not with ulcerative colitis disease. In terms of clinical remission in the treatment of IBD, more importance should be given to mucosal healing, as it predicts durable complete remission [44]. Several studies have shown that the HBi has low specificity and does not correlate well with endoscopic or histological disease activity in patients with CD [45,46]. A significant proportion of patients who report clinical remission have mucositis [47]. The Mayo score is considered more reliable in assessing disease activity in patients with UC, as it includes endoscopic assessment and clinical assessments by physicians, whereas the HBi gives more weight to subjective symptoms of patients with CD [48]. It is worth noting that FC is now widely recommended as a sign of intestinal mucosal healing [49]. Low FC was shown to predict sustained clinical remission in patients with IBD [50]. In our analysis, only one study showed a significant reduction in FC levels with LFD [20]. It should be noted, however, that in most studies, participants remained in clinical remission. It remains to be determined whether the small reduction in HBi score may indeed reflect variability in disease activity. Furthermore, it remains unclear whether low disease activity limits the potential of an LFD to further reduce inflammation. Therefore, further studies with larger sample sizes and more comprehensive analysis are warranted to confirm the results.

Only one study examined the effectiveness of LFD in a condition other than IBS or IBD, and that was PPI-resistant GERD [16]. The authors noted that LFD significantly reduced only the severity of gastrointestinal symptoms and those related to reflux. Reflux symptoms also decreased in the group using usual dietary advice and lifestyle modifications, but there were no differences between the groups. Plaidum et al. [51] conducted a randomized crossover study with overlapping GERD-IBS (non-constipation) to evaluate the effects of rice noodle (part of a low-FODMAP diet) vs. wheat noodle (part of a high-FODMAP diet) meals for breakfast and lunch on postprandial TLESR (Transient Lower Esophageal Relaxations) and GERD/GI symptoms. The authors noted that wheat noodle meals induced a higher frequency of TLESRs, a higher GERD, and higher GI symptom scores (like regurgitation, bloating, and belching) than rice noodle meals. However, it is worth taking into account that the group of people studied was very small and consisted of eight people. On the other hand, a meta-analysis from 2024 examined the effectiveness of various dietary interventions in patients with GERD [52]. Only two interventions concerned the FODMAP diet, but analysis could not be performed for them, which indicates that there is still too little research in this area.

The FODMAP diet may have a positive effect on reducing gastrointestinal symptoms and quality of life in various diseases, but various limitations and consequences related to its use and its impact on other factors/parameters should be taken into account. The low-FODMAP diet restricts short-chain carbohydrates that are osmotically active and rapidly fermented by colonic microbiota. Their malabsorption leads to increased luminal water and gas production, contributing to visceral hypersensitivity and gastrointestinal symptoms. By reducing fermentable substrate availability, the diet decreases intraluminal distension and symptom severity. A full LFD protocol should include three phases: restriction, reintroduction, and personalization [53]. In most randomized controlled trials, the authors focus only on the restriction phase (usually lasting 3–6 weeks), not including reintroduction of products to the patient’s diet, as well as subsequent monitoring of symptoms, disease activity or quality of life, e.g., in the period of 6 months or one year after the implementation of the dietary intervention. Such a procedure could prevent the occurrence of nutritional deficiencies, deterioration of nutritional status, or relapse/exacerbation of the disease. Retrospective studies had longer follow-up periods, up to 16 months [32,54]. Considering that diseases such as IBS or IBD are chronic diseases, the short duration of most scientific studies raises questions about the durability of the clinical benefits of the low-FODMAP diet. Studies lasting more than 12 months have shown that the LFD should be continued for at least three months to be most effective. The greatest improvement in symptoms was observed in patients who fully adhered to the LFD [55]. However, the LFD can be cumbersome to follow because of the constant need for support from a trained dietitian and the careful restriction of otherwise healthy foods. Furthermore, patients often follow the diet alone, without the help of trained support staff, which may contribute to poor adherence to the LFD, resulting in a lack of patient benefit and poorer outcomes. According to Tuck et al. [56], only 30% of patients consulted a dietitian for advice on the LFD. However, in patients with disorders of gut–brain interaction (DGBI), dietitian-led consultations were found to lead to high levels of clinical effectiveness, and brochures were the least acceptable methods of delivering education [57]. The identification of appropriate recipes is essential, as some patients perceive the diet to be overly bland. Moreover, prior research indicates that higher educational attainment and working fewer than 35 h per week are factors that contribute to an improved understanding of and adherence to the diet. Additionally, individuals with limited financial resources may encounter difficulties in maintaining adherence, even for short periods. These challenges are further compounded by the lack of standardization in food labeling, as FODMAP content is not commonly reported in most countries [58].

Another very important aspect is the impact of LFD use on the state of the intestinal microbiota. Research in this area is inconsistent. Several investigators reported a relative decrease in total bacterial abundance [59] and bacteria thought to be beneficial to the GI tract [25,60]. For example, some studies found reduced *Bifidobacterium* species [7,8,10,61], a genus that harbors many probiotic strains, the depletion of which might be harmful in the long term, and increases in potentially harmful species such as *Porphyromonadaceae* [9]. In addition, some studies also found a loss of SCFAs producers, such as *Clostridiales*, *Bacteroides*, *Prevotella*, and *Actinobacteria*, and an increase in the non-saccharolytic taxon *Bilophila* [7,10]. Some of these changes may be reversible with the reintroduction of FODMAPs [62] and/or with probiotic/prebiotic use [8,62]. While prebiotic supplementation did not prevent the loss of bifidobacteria species, the probiotic supplementation maintained *Bifidobacterium* spp. at levels comparable to the baseline [54]. In turn, the authors of the latest meta-analysis from 2025 suggest that a low-FODMAP diet may have a positive effect on the regulation of intestinal microbiota, but this effect seems to be less pronounced in patients with IBS [58]. It is important to note that this meta-analysis looks at overall changes in the gut microbiota, and the specific bacterial taxa affected by low-FODMAP-focused diets may vary. The heterogeneity observed and the results of the subgroup analysis highlight the complexity of this relationship and warrants careful consideration. Furthermore, in another review from 2025, the authors emphasize the importance of a personalized approach to dietary management in IBS, taking into account individual differences in microbiome responses [63]. A more detailed analysis of taxonomic changes, also in individuals with other gastrointestinal diseases, is an important area of future research.

The findings of this systematic review suggest that despite heterogeneity in study design and outcome measures, an LFD may provide short-term relief of gastrointestinal symptoms—particularly abdominal pain and bloating—in selected patient populations, especially those with IBS. However, it is important to interpret these results with caution. The LFD is not intended as a universal or long-term dietary strategy for all patients with gastrointestinal disorders. Rather, it should be considered a short-term, structured intervention, particularly in individuals whose symptoms significantly impair quality of life. Long-term adherence to this diet without appropriate reintroduction phases may negatively impact gut microbial diversity, nutritional adequacy, and dietary variety. Indeed, several studies have indicated potential reductions in beneficial commensal bacteria following extended restriction phases, raising concerns about microbiota-related consequences and possible micronutrient deficiencies. Furthermore, while the diet is frequently recommended in clinical practice for patients with SIBO, our review did not identify any randomized controlled trials demonstrating its efficacy in this specific subgroup. This gap highlights the need for more targeted and robust clinical research before generalizing the use of the LFD approach beyond its currently evidence-supported indications. Taken together, these findings underscore the importance of applying the LFD selectively and under the supervision of trained healthcare professionals, with careful attention to the reintroduction process and monitoring of nutritional status.

### Strengths and Limitations

In the context of the low-FODMAP diet, its use in IBS is most often mentioned, but in our review, we focused on expanding the scope of database searches to include other diseases and disorders of the digestive system. A key strength of this review lies in its adherence to the PRISMA guidelines for reporting systematic reviews [11], as well as the comprehensive database searches and rigorous quality assessments conducted independently by two researchers. The inclusion criteria were explicitly defined and strictly followed throughout this study. The analyzed studies were conducted across various populations and countries. An important inclusion criterion is that we only analyzed studies in which information on diets was provided by trained individuals, which excluded the risk of following a low-FODMAP diet inconsistently with its assumptions. We also only analyzed studies that compared the effect of the low-FODMAP diet without any other dietary intervention conducted in the same group or the implementation of dietary supplements/probiotics/prebiotics, etc., which could distort the reliable assessment of the effect of the low-FODMAP diet alone.

We acknowledge several limitations in both this paper and the studies included in this review. In our review, we did not consider the effect of the LFD on other important parameters such as changes in microbiota composition, nutritional status, or anthropometric parameters. All of the above factors may affect the validity of this type of intervention. The analysis was based on a relatively small number of studies, the majority of which exhibited significant methodological limitations or a moderate risk of bias. The analysis was based on a relatively small number of studies, the majority of which exhibited significant methodological limitations or a moderate risk of bias. The included studies differed in the questionnaires used and the way they assessed gastrointestinal symptoms and quality of life; data were sometimes presented in different ways. Some studies lacked specific results (e.g., between-group comparisons) and reported incomplete statistical analyses (e.g., no *p*-values). These factors often hinder or preclude direct comparison of results and the conduction of a meta-analysis. Moreover, the included studies were characterized by relatively small sample sizes, and the interventions typically did not exceed a duration of six weeks. The articles concerned the use of the LFD in only three gastrointestinal diseases, and its implementation in people with SIBO is increasingly discussed, which indicates a new direction of research.

Although this review synthesizes current evidence qualitatively, the substantial heterogeneity in study designs, outcome measures, and reporting formats precluded a formal meta-analysis. The interpretation of findings was substantially challenged by differences in the design and delivery of dietary interventions—such as the degree of restriction in low-FODMAP protocols, variations in comparator diets (e.g., conventional diet or other structured plans), and inconsistencies in intervention duration and follow-up—which limited direct comparability. Moreover, clinical outcomes were assessed using diverse tools measuring symptom severity, quality of life, or stool patterns, while blinding was rarely feasible in dietary trials, introducing potential bias. Given these methodological disparities, we strongly emphasize the need not only for more randomized controlled trials but also for better-conducted and more standardized studies. Future research should prioritize uniformity in the structure and delivery of dietary interventions, adoption of standardized outcome measures, and clear definitions of clinical endpoints. Additionally, robust reporting on adherence, implementation protocols, and nutritional adequacy is essential to enhance reproducibility and clinical applicability. Focused systematic reviews on narrowly defined populations and standardized endpoints may then allow for quantitative synthesis, providing more robust estimates of the low-FODMAP diet’s effectiveness across specific gastrointestinal conditions. Addressing these methodological gaps will enable a more meaningful synthesis of findings and improve our understanding of the true efficacy and safety of the low-FODMAP diet in managing gastrointestinal disorders.

## 5. Conclusions

In conclusion, this systematic review demonstrates that a low-FODMAP diet appears to be effective in reducing gastrointestinal symptoms—particularly the severity and frequency of abdominal pain and bloating—and in improving health-related quality of life in individuals with irritable bowel syndrome (IBS) when compared to control diets. The evidence for its efficacy in patients with inflammatory bowel disease (IBD) is less consistent, with potential benefits limited mainly to functional gastrointestinal symptoms rather than disease activity. For gastroesophageal reflux disease (GERD), the available evidence is very limited, and current data do not allow for definitive conclusions regarding its effectiveness.

However, these findings should be interpreted with caution due to considerable heterogeneity among the included studies in terms of study design, populations, outcome measures, and intervention protocols. Importantly, the low-FODMAP diet is not universally necessary and should not be recommended to all patients with gastrointestinal complaints. Rather, it may serve as a short-term therapeutic option in selected individuals with significant symptom burden.

Given the potential for negative long-term consequences, such as nutritional deficiencies and alterations in gut microbiota composition, clinical implementation of the low-FODMAP diet should always occur under the supervision of qualified healthcare professionals, preferably dietitians specialized in gastrointestinal disorders. Furthermore, greater attention should be paid to basic dietary and hygiene recommendations as part of a comprehensive care plan. In some individuals, particularly those with milder symptoms, improvements in food hygiene practices and the reduction or elimination of ultra-processed foods and high-sugar products may be sufficient to achieve symptom relief, without the need to initiate a restrictive dietary protocol such as the low-FODMAP diet.

Future research should prioritize large-scale randomized controlled trials with standardized outcome measures and longer follow-up periods. Personalized dietary approaches and investigation of response predictors are also warranted to optimize therapeutic efficacy and patient safety.

## Figures and Tables

**Table 1:** Characteristics of the studies included in this systematic review.

First Author, Year (Country)	Disease	Total Case/Controls	Age Case/Controls (Years; Mean ± SD)	Study Duration	Characteristics of Diets
Böhn et al., 2015 (Sweden) [21]	IBS	38/37	44 (18–69)/41 (18–68) *	4 weeks	**LFD:** Defined by the restriction of foods containing fermentable oligosaccharides, monosaccharides, disaccharides, and polyols. Patients were provided with a pamphlet containing detailed guidance on foods to avoid as well as alternative food options suitable for consumption. **Diet frequently recommended for patients with IBS:** Emphasizes the timing and manner of food consumption rather than solely focusing on specific food choices. It is based on dietary recommendations from NICE [26] and the BDA [27].
Eswaran et al., 2017 (US) [25]	IBS-D	50/42	41.6 ± 14.7/43.8 ± 15.2	4 weeks	**LFD:** Defined by the restriction of foods containing fermentable oligosaccharides, monosaccharides, disaccharides, and polyols. The instruction followed published materials from Monash University [28]; however, participants received educational materials developed by the University of Michigan. **mNICE:** Modified guidance from the NICE. Patients were advised to consume small, frequent meals, avoid trigger foods, and limit excessive alcohol and caffeine intake. The mNICE guidelines provided to study participants did not specifically exclude foods containing FODMAPs.
Liu et al., 2024 (China) [19]	IBS	15/18	34.9 ± 12.5/35 ± 9.8	4 weeks	**LFD:** Patients are advised to limit their consumption of foods high in FODMAPs as specified in the published literature and in a mobile application produced by Monash University [29]. **Conventional diet:** Patients maintained their habitual Chinese diet while adhering to conventional dietary recommendations, which included consuming regular meals, ensuring adequate water intake, and reducing the consumption of fat, alcohol, caffeine, spicy foods, and other foods that may exacerbate intestinal symptoms.
Ostrowska et al., 2021 (Poland) [14]	IBS-M	26/21/26	42.7 ± 16.7/40.6 ± 14.5/41.7 ± 13.4	8 weeks	**LFD:** Patients received personalized dietary guidance, along with educational materials containing a 7-day sample menu and a table outlining recommended and contraindicated foods in the FODMAP diet. **IgG-based elimination–rotation diet:** Patients underwent IgG antibody titer testing for specific foods to identify potential food hypersensitivities. Based on the test results, each patient received guidance on an elimination–rotation diet and an exemplary menu. Foods testing positive for IgG antibodies were excluded from the diet, while all IgG-negative foods were permitted within the rotation diet. **Control diet:** Patients were advised to follow dietary treatment as recommended by their attending gastroenterologist. They were provided with an easily digestible diet, which was a modified version of balanced nutrition for healthy individuals, meeting the same energy requirements and providing equivalent nutrient intake as a standard diet. During episodes of diarrhea, patients adhered to an easily digestible diet with restricted intake of fat and the insoluble fraction of dietary fiber. During periods of constipation, patients were advised to increase their dietary fiber intake.
Patcharatrakul et al., 2019 (Thailand) [24]	IBS	30/32	50 ± 13.7/52 ± 14	4 weeks	**SILFD:** High-FODMAP items potentially worsening symptoms were identified from a 7-day food diary. Then patients avoided these items and modified their menus with commonly available low-FODMAP alternatives. A sample menu using low-FODMAP foods was included in the pamphlets provided to the patients. **BRD:** It recommended reducing foods commonly associated with gas, bloating, or abdominal pain, including fruits, vegetables, nuts, beans, and garlic, and avoiding large meals. The term FODMAP was not mentioned during the advice.
Rej et al., 2022 (UK) [15]	IBS-D or IBS-M	33/33/33	35 ± 12/40 ± 15/36 ± 13	4 weeks	**LFD:** Defined by the limiting of foods containing fermentable oligosaccharides, monosaccharides, disaccharides, and polyols. **TDA:** Based on dietary recommendations from NICE and the BDA [26,30,31]. Its principles include having regular meals, never eating too little/too much, maintaining adequate hydration, reducing the intake of alcohol/caffeine/fizzy drinks and fatty/spicy/processed foods, fresh fruit to a maximum of 3 per day, fiber and other commonly consumed gas-producing foods, and addressing any perceived food intolerances. **GFD:** Defined by the exclusion of gluten.
Russo et al., 2022 (Italy) [17]	IBS-D	21/21	N/A	12 weeks	**LFD:** Defined by the limiting of foods containing fermentable oligosaccharides, monosaccharides, disaccharides, and polyols. Participants received a structured weekly menu outlining three main meals (breakfast, lunch, dinner) and two snacks (morning and afternoon). They were provided with a booklet specifying permitted, restricted, and limited foods, based on Monash University classifications and FODMAP subgroup cut-off values [32,33,34], and a leaflet outlining where to purchase specific products. Guidance was provided on maintaining sufficient fiber intake and preparing meals without high-FODMAP ingredients like onions and garlic. Alcohol consumption was discouraged. **TBD:** Each patient had to consume flour, bread, breakfast biscuits, taralli (local salty biscuits), and pasta prepared exclusively with Tritordeum. A controlled diet was provided to each patient. The daily menu was breakfast, mid-morning snacks, lunch, afternoon snacks, and dinner.
Tunali et al., 2024 (Turkey) [18]	All subtypes of IBS	51/70	37.9 ± 9.87/35.94 ± 10.13	6 weeks	**LFD:** Involved a restricted intake of foods containing fermentable oligosaccharides, monosaccharides, disaccharides, and polyols. Patients received a pamphlet outlining restricted foods and suitable alternatives. The guidelines were developed based on previously published resources from the ACG [35]. **PD:** It was designed based on AI-recommended foods derived from microbiome analysis results [36]. Approximately 300 foods were assessed on a scale of 0 to 10 for their impact on microbiome modulation. Foods scoring 0–3 were classified as avoidable, those scoring 4–7 as suitable for dietary diversification, and those scoring 8–10 as essential. The dietitian primarily incorporated foods with scores between 4 and 10, prioritizing high-scoring fruits. Low-scoring foods were not recommended. In the initial weeks, raw greens and legumes were restricted, with high-scoring foods gradually introduced into the diet.
Zahedi et al., 2018 (Iran) [23]	IBS-D	50/51	37.6 ± 11.09/37.43 ± 13.27	6 weeks	**LFD:** The diet provided less than 0.5 g per meal of fermentable oligosaccharides, monosaccharides, disaccharides, and polyols [37]. Patients received a pamphlet detailing suitable and unsuitable foods, alternative options, a shopping guide, strategies for dining out, and guidance on preparing meals without onion and garlic. **GDA:** Incorporated dietary recommendations from the BDA [30], including limiting caffeine, alcohol, spicy foods, high-fat foods, and carbonated beverages. Additional guidelines emphasized consuming small, frequent meals, eating slowly in a calm environment, and avoiding chewing gum and sweeteners containing polyols.
Zhang et al., 2021 (China) [7]	IBS-D	54/54	42.4 ± 10.7/44.6 ± 14.6	3 weeks	**LFD:** Patients were advised to eliminate high-FODMAP foods based on guidelines from the published literature and a Monash University mobile application [29]. **TDA:** Patients were instructed to reduce the intake of fatty and spicy foods, coffee, and alcohol and to follow a regular meal pattern of three meals per day. They were encouraged to avoid overeating or undereating and to eat calmly, chewing food thoroughly [21]. FODMAP-containing foods were not explicitly excluded.
Bodini et al., 2019 (Italy) [20]	IBD in remission or with mild disease activity	26/29	41 (34–48)/47 (44–57) *	6 weeks	**LFD:** Defined by the restriction of foods containing fermentable oligosaccharides, monosaccharides, disaccharides, and polyols. **SD:** Contained a usual FODMAP amount. All patients from both groups were encouraged to have three main meals (breakfast, lunch, and dinner) with varying types and portions of food, along with two snacks throughout the day. At each main meal, the dietitian offered patients the option to choose from at least three different menu options, each with the same caloric and FODMAP content. Additionally, patients received informational leaflets on meal preparation.
Cox et al., 2020 (UK) [13]	Quiescent IBD	27/25	33 ± 11/40 ± 13	4 weeks	**LFD:** Defined by the restriction of foods containing fermentable oligosaccharides, monosaccharides, disaccharides, and polyols. **Control (sham) diet:** It was designed to offer patients an exclusion diet of comparable intensity and burden to the LFD while maintaining consistent nutrient, fiber, and FODMAP intake levels.
Pedersen et al., 2017 (Denmark) [22]	IBD in remission or with mild-to-moderate disease and coexisting IBS-like symptoms	37/41	40 (20–70)/41 (24–69) *	6 weeks	**LFD:** Defined by the restriction of foods containing fermentable oligosaccharides, monosaccharides, disaccharides, and polyols. **ND:** Patients were requested to follow an unchanged habitual diet.
Rivière et al., 2021 (France) [16]	Symptomatic PPI refractory GERD	16/15	47 (37–50)/44 (40–52) *	4 weeks	**LFD:** The diet was developed based on previously published studies on IBS patients [38], aiming to limit total FODMAP intake to less than 3 g/day. **UDA:** Usual dietary advice and lifestyle modifications include reducing the intake of high-fat foods, alcohol, caffeine, and tobacco, avoiding overeating, maintaining an upright position for at least two hours after meals, elevating the head of the bed, and refraining from eating within two hours of bedtime.

* Median (range); ACG—American College of Gastroenterology; BDA—British Dietetic Association; BRD—brief advice on a commonly recommended diet; FODMAP—fermentable oligosaccharides, disaccharides, monosaccharides, and polyols; GDA—general dietary advice; GERD—gastroesophageal reflux disease; GFD—gluten-free diet; IBD—inflammatory bowel disease; IBS—irritable bowel syndrome; IBS-M—irritable bowel syndrome mixed type; IBS-D—irritable bowel syndrome with diarrhea; N/A—not detailed results available; LFD—low-FODMAP diet; mNICE—modified diet recommended by the NICE; ND—normal diet; NICE—National Institute for Health and Care Excellence; PD—microbiome-based artificial-intelligence-assisted personalized diet; PPI—proton pump inhibitor; SD—standard diet; SILFD—structural individual low-FODMAP dietary advice; TBD—Tritordeum-based foods; TDA—traditional dietary advice; UDA—usual dietary advice.

**Table 2:** Parameters in a case and control group before and after intervention among participants with IBS.

Authors	Parameter [Unit]	Case Group Baseline vs. After Intervention	*p*-Value	Control Group Baseline vs. After Intervention	*p*-Value	*p*-Value After Intervention vs. Control
Böhn et al. [21]	Total IBS-SSS score, mean ± SD	324 ± 69 vs. 246 ± 127	<0.001	302 ± 61 vs. 236 ± 78	<0.001	NS
Eswaran et al. [25]	Total IBS-QOL score, (95% CI)	53.4 vs. 69.3 (10.9 to 20.8)	<0.05	54.3 vs. 59.4 (0.56 to 9.46)	<0.05	<0.05
	HADS: Anxiety scores, mean (95% CI)	9.13 vs. 7.73 (−2.10 to −0.59)	<0.05	9.31 vs. 9.54 (−0.64 to 1.10)	NS	<0.05
	HADS: Depression scores, mean (95% CI)	4.6 vs. 3.68 (−1.57 to −0.28)	<0.05	5.51 vs. 4.89 (−1.42 to 0.17)	NS	NS
	Absenteeism (work time missed), mean ± SD	5.59 ± 14 vs. 5.10 ± 12	NS	1.78 ± 5 vs. 6.47 ± 21	NS	NS
	Presenteeism (impairment while working), mean ± SD	38.4 ± 23 vs. 26.3 ± 23	NS	35.9 ± 25 vs. 33.04 ± 23	NS	NS
	Productivity loss (overall work impairment), mean ± SD	40.3 ± 24 vs. 28.3 ± 25	NS	37.5 ± 24± vs. 34.5 ± 23	NS	NS
	Activity impairment, mean ± SD	52.6 ± 22 vs. 29.7 ± 24	N/A	52.8 ± 25 vs. 43.3 ± 28	N/A	NS
	Sleep quality, mean ± SD	5.34 ± 2 vs. 4.38 ± 2	<0.05	5.14 ± 2 vs. 4.61 ± 2	N/A	NS
	Fatigue, mean ± SD	5.06 ± 2 vs. 4.19 ± 2	<0.05	5.44 ± 2 vs. 4.9 ± 3	N/A	NS
	Overall sleep QOL, mean ± SD	7.32 ± 2 vs. 6.42 ± 2	<0.05	7.68 ± 2 vs. 7.49 ± 2	NS	NS
Liu et al. [19]	Total IBS-SSS score, mean ± SD	208.56 ± 57.26 vs. 116 ± 51.37	<0.05	202.80 ± 90.97 vs. 232.20 ± 113.74	NS	<0.05
Ostrowska et al. [14]	Idiopathic abdominal pain, *n* (%)	G1-FM: 15 (58) vs. 11 (42) G2-IP: 16 (76) vs. 2 (10)	NS <0.001	16 (62) vs. 14 (54)	NS	N/A
	Abdominal pain after a meal, *n* (%)	G1-FM: 11 (42) vs. 6 (23) G2-IP: 14 (67) vs. 2 (10)	NS <0.001	14 (54) vs. 12 (46)	NS	N/A
	Abdominal pain during defecation, *n* (%)	G1-FM: 5 (19) vs. 2 (8) G2-IP: 9 (43) vs. 1 (5)	NS 0.008	6 (23) vs. 6 (23)	NS	N/A
	Sensation of incomplete defecation, *n* (%)	G1-FM: 13 (50) vs. 10 (39) G2-IP: 13 (62) vs. 2 (10)	NS 0.001	14 (54) vs. 15 (58)	NS	N/A
	Difficulty to defecate (constipations), *n* (%)	G1-FM: 11 (42) vs. 7 (27) G2-IP: 14 (67) vs. 4 (19)	NS 0.002	19 (73) vs. 17 (65)	NS	N/A
	Bloating, *n* (%)	G1-FM: 22 (85) vs. 7 (27) G2-IP: 19 (91) vs. 2 (10)	<0.001 <0.001	24 (92) vs. 22 (85)	NS	N/A
	Gurgling sensation, *n* (%)	G1-FM: 17 (65) vs. 4 (15) G2-IP: 18 (86) vs. 2 (10)	<0.001 <0.001	21 (81) vs. 19 (73)	NS	N/A
	Gastric fullness, *n* (%)	G1-FM: 15 (58) vs. 3 (12) G2-IP: 19 (91) vs. 2 (10)	<0.001 <0.001	22 (85) vs. 19 (73)	NS	N/A
Patcharatrakul et al. [24]	Total IBS-SSS score 0–100, mean ± SD	61.2 ± 21 vs. 38.5 ± 20	<0.001	56.3 ± 17.8 vs. 53.5 ± 19.2	NS	0.006
Rej et al. [15]	Total IBS-SSS score, mean ± SD	LFD: 311 ± 80 vs. 148 ± 87 GFD: 299 ± 73 vs. 180 ± 89	N/A	330 ± 74 vs. 199 ± 93	N/A	NS
	Total IBS-QOL score, mean ± SD	LFD: 51 ± 21 vs. 61 ± 24 GFD: 60 ± 26 vs. 65 ± 26	N/A	52 ± 18 vs. 55 ± 21	N/A	NS
	HADS: Anxiety scores, mean ± SD	LFD: 10.6 ± 5.4 vs. 8.9 ± 5.1 GFD: 9.8 ± 5.4 vs. 8.4 ± 4.9	N/A	9.5 ± 4.4 vs. 9.5 ± 4.4	N/A	NS
	HADS: Depression scores, mean ± SD	LFD: 7.6 ± 4.5 vs. 6.5 ± 5.2 GFD: 6.7 ± 4.6 vs. 6.4 ± 5	N/A	6.8 ± 3.3 vs. 7.6 ± 3.5	N/A	0.03
	PHQ-12 score, mean (SD)	LFD: 8.5 ± 4 vs. 7.7 ± 3.7 GFD: 8.4 ± 3.6 vs. 7.9 ± 4.2	N/A	9.6 ± 4.7 vs. 8.7 ± 3.7	N/A	NS
Russo et al. [17]	Total IBS-SSS score, (95% CI)	N/A vs. −132.1 (−74.9 to −189.4), ↓	<0.0001	N/A vs. −130.5 (−73.2 to −187.7), ↓	<0.0001	NS
Tunali et al. [18]	Total IBS-SSS score, mean ± SD	276.76 ± 90.15 vs. 176.86 ± 111.09	<0.001	314.42 ± 92.7 vs. 210.64 ± 130.63	<0.001	NS
	Total IBS-QOL score, mean ± SD	42.65 ± 19.82 vs. 55.08 ± 23.62	<0.001	45.55 ± 22.06 vs. 55.79 ± 21.85	<0.001	NS
	HADS: Anxiety scores, mean ± SD	10.74 ± 3.95 vs. 7.86 ± 4.07	<0.001	10.27 ± 4.22± vs. 8.15 ± 3.37	<0.001	NS
	HADS: Depression scores, mean ± SD	8.33 ± 4.36 vs. 5.72 ± 4.35	<0.001	7.57 ± 4.35 vs. 6.22 ± 4.10	0.02	NS
Zahedi et al. [23]	Total IBS-SSS score, mean ± SD	263.75 ± 91.25 vs. 108 ± 63.82	<0.001	252.5 ± 85.51 vs. 149.75 ± 51.39	<0.001	<0.001
	Total IBS-QOL score, mean ± SD	51.03 ± 17.48 vs. 43.73 ± 8.78	<0.001	50.3 ± 16.81 vs. 44.95 ± 9.19	0.001	NS
Zhang et al. [7]	Total IBS-SSS score, mean ± SD	244.6 ± 76.3 vs. N/A	N/A	231.2 ± 69.4 vs. N/A	N/A	NS
	Total IBS-QOL score, mean ± SD	42.2 ± 25.1 vs. 23 ± 20.4	<0.001	36.5 ± 21.9 vs. 18.6 ± 17.6	<0.001	NS
	GAD-7, mean ± SD	5.9 ± 4.9 vs. 3.7 ± 3.9	0.001	6.6 ± 4.7 vs. 3.4 ± 3.9	<0.001	NS
	PHQ-9, mean ± SD	4.9 ± 4.2 vs. 3.5 ± 4	0.012	6 ± 4.3 vs. 3.7 ± 3.8	<0.002	NS

GAD-7—General Anxiety Disorder; GFD—gluten-free diet; G1-FM—group 1 low-FODMAP diet; G2-IP—group 2 immunoglobulin G (IgG)-based elimination–rotation diet; HADS—Hospital Anxiety and Depression Scale; IBS-QOL—Irritable Bowel Syndrome Quality Of Life; IBS-SSS—Irritable Bowel Syndrome Symptom Severity Score; LFD—low-FODMAP diet; NS—not statistically significant; N/A—not detailed results available; PHQ-9—Patient Health Questionnaire-9; PHQ-12—Patient Health Questionnaire-12; QOL—quality of life; SD—standard deviation; ↓ = decrease in scores compared to baseline.

**Table 3:** Parameters in a case and a control group before and after intervention among participants with IBD.

Authors	Parameter [Unit]	Case Group Baseline vs. After Intervention	*p*-Value	Control Group Baseline vs. After Intervention	*p*-Value	*p*-Value After Intervention vs. Control
Bodini et al. [20]	HBi, median (IQR)	4 (3–5) vs. 3 (2–3)	0.024	3 (3–3) vs. 3 (2–4)	NS	NS
	Mayo score, median (IQR)	2 (2–3) vs. 1 (1–3)	NS	2 (2–3) vs. 2 (1–2)	NS	NS
	Fecal calprotectin [mg/kg], median (IQR)	76.6 (50–286.3) vs. 50 (50.6–81)	0.004	91 (50.6–143.6) vs. 87 (50–235.6)	NS	NS
	IBDQ, median (IQR)	166 (139–182) vs. 177 (155–188)	0.05	181 (153–197) vs. 166 (153–200)	NS	NS
Cox et al. [13]	Total IBS-SSS score, median (SEM)	222 (76) vs. 158 (12)	N/A	227 (81) vs. 190 (13)	N/A	NS
	IBS-SSS 50% reduction, *n* (%)	N/A vs. 9 (33)	N/A	N/A vs. 1 (4)	N/A	0.012
	Adequate relief, *n* (%)	N/A vs. 14 (52)	N/A	N/A vs. 4 (16)	N/A	0.007
	Stool frequency (per d), mean (SEM)	1.8 (1.3) vs. 1.7 (0.1)	N/A	2.1 (1.0) vs. 2.1 (0.1)	N/A	0.012
	UK IBDQ, median (SEM)	N/A vs. 81.9 (1.2)	N/A	N/A vs. 78.3 (1.2)	N/A	0.042
	HBi, median (SEM)	N/A vs. 3.2 (0.4)	N/A	N/A vs. 3.4 (0.5)	N/A	NS
	Mayo score, median (SEM)	N/A vs. 0.2 (0.2)	N/A	N/A vs. 0.2 (0.2)	N/A	NS
	Fecal calprotectin [µg/g], median (IQR)	54.8 (84.8) vs. 53.3 (84.8)	NS	70.9 (117.3) vs. 66.9 (106.4)	NS	NS
Pedersen et al. [22]	Total IBS-SSS score in all participants, median (IQR)	210 (190–270) vs. 115 (33–169)	<0.001	245 (180–320) vs. 170 (91–288)	<0.001	0.02
	Total IBS-QOL score in all participants, median (IQR)	73 (19–99) vs. 78 (51–65)	<0.01	77 (30–98) vs. 81 (26–99)	NS	NS
	Total SIBDQ score in all participants, median (IQR)	47 (42–55) vs. 60 (51–65)	<0.01	50 (40–57) vs. 50 (39–60)	NS	<0.01
	HBi, median (IQR)	7 (3–8) vs. 3 (1–5)	0.05	5 (4–11) vs. 6 (3–9)	NS	NS
	SCCAI, median (IQR)	3 (1–4) vs. 1 (0–3)	0.04	2 (1–4) vs. 2 (1–4)	NS	0.02
	Fecal calprotectin [µg/g], (95% CI)	65 (37 to 113) vs. 53 (30 to 93)	NS	44 (23 to 83) vs. 46 (27 to 81)	NS	NS

HBi—Harvey–Bradshaw index (assesses the activity of Crohn’s disease); IBDQ—Inflammatory Bowel Disease Health-Related Quality of Life Questionnaire; IBS-QOL—Irritable Bowel Syndrome Quality of Life; IBS-SSS—Irritable Bowel Syndrome Symptom Severity Score; IQR—interquartile range; Mayo score—assesses the activity of ulcerative colitis disease; NS—not statistically significant; N/A—not detailed results available; SEM—estimated marginal mean; SCCAI—Simple Clinical Colitis Index; SIBDQ—Short Inflammatory Bowel Syndrome Quality Of Life Questionnaire; UK IBDQ—United Kingdom version of the IBDQ.

**Table 4:** Parameters in a case and a control group before and after intervention among patients with symptomatic PPI refractory GERD.

Authors	Parameter [Unit]	Case Group Baseline vs. After Intervention	*p*-Value	Control Group Baseline vs. After Intervention	*p*-Value	*p*-Value After Intervention vs. Control
Rivière et al. [16]	Total RDQ score, median (IQR)	4.3 (3.8–4.8) vs. 3.4 (3.0–4.3)	0.002	4.2 (3.6–4.8) vs. 3.9 (3.2–4.7)	0.002	NS
	Total IBS-SSS score, median (IQR)	245 (135–324) vs. 166 (93–203)	0.04	290 (157–319) vs. 191 (168–274)	NS	NS
	Total GIQLI, median (IQR)	73 (61–85) vs. 85 (72–94)	NS	83 (69–89) vs. 83 (64–95)	NS	NS
	Total reflux events, median (IQR)	59 (47–75) vs. 46 (35–61)	N/A	67 (58–87) vs. 51 (28–99)	N/A	NS
	Total acid exposure, median (IQR)	1.1 (0.2–2.4) vs. 0.9 (0.0–1.9)	N/A	1.1 (0.3–2.3) vs. 1.0 (0.2–2.0)	N/A	NS
	Total bolus exposure, median (IQR)	2.0 (1.6–3.3) vs. 1.4 (1.1–1.7)	N/A	2.8 (1.7–3.8) vs. 2.9 (1.9–6.2)	N/A	NS
	Positive symptomatic association (%)	7 (44) vs. 5 (31)	N/A	9 (60) vs. 12 (80)	N/A	NS

GIQLI—Gastrointestinal Quality of Life Index; IBS-SSS—Irritable Bowel Syndrome Symptom Severity Score; IQR—interquartile range; RDQ—Reflux Disease Questionnaire; NS—not statistically significant; N/A—not detailed results available.

## Supplementary Materials

The following supporting information can be downloaded at: https://www.mdpi.com/article/10.3390/nu17122045/s1, Table S1: Literature searching strategy; Table S2: Results of the quality assessment of randomized studies using the CASP Randomised Controlled Trial Standard Checklist—Sections A and B.
