# Supplementary material for: Efficacy of a Low-FODMAP Diet on the Severity of Gastrointestinal Symptoms and Quality of Life in the Treatment of Gastrointestinal Disorders—A Systematic Review of Randomized Controlled Trials
Source: Nutrients. 2025 Jun 19;17(12):2045. doi: 10.3390/nu17122045 (PMC12196412; doi:10.3390/nu17122045)
Supplement: Supplementary file 1 [file nutrients-17-02045-s001.zip › nutrients-3688356-supplementary.pdf]

**Table S1.** Literature searching strategy

| Search number | Search term | PubMed | Scopus | Web of Science |
|---|---|---|---|---|
| #1 | (randomized controlled) OR (randomized controlled trial) OR (randomized controlled trials) OR (randomised controlled) OR (randomised controlled trial) OR (randomised controlled trials) | 1,008,375 | 4,441,403 | 743,111 |
| #2 | (FODMAP) OR (FODMAPS) OR (low FODMAP diet) OR (low FODMAP) OR (fermentable oligosaccharides, disaccharides, monosaccharides and polyols) OR (fermentable, poorly absorbed, short-chain carbohydrates) OR (oligosaccharides) OR (fructans) OR (fructo-oligosaccharides) OR (oligo-fructose) OR (inulin) OR (galactans) OR (galacto-oligosaccharides) OR (disaccharides) OR (lactose) OR (monosaccharides) OR (fructose) OR (polyols) OR (sorbitol) OR (xylitol) OR (mannitol) OR (maltitol) OR (isomalt) | 644,410 | 1,151,055 | 284,266 |
| #3 | (gastrointestinal disorders) OR (gastrointestinal diseases) OR (gastrointestinal symptoms) OR (functional gastrointestinal disorders) OR (intestinal diseases) OR (colitis) OR (enteritis) OR (inflammatory intestinal disease) OR (inflammatory enteropathy) OR (bowel inflammation) OR (intestinal inflammation) OR (ileitis) OR (colonitis) OR (Crohn's disease) OR (ulcerative colitis) OR (diverticular disease) OR (diverticulitis) OR (diverticulosis) OR (colon cancer) OR (colorectal cancer) OR (colonic polyps) OR (irritable bowel syndrome) OR (irritable colon) OR (spastic colon) OR (nervous colon) OR (mucous colitis) OR (spastic bowel) OR (small intestinal bacterial overgrowth) | 1,603,867 | 4,084,417 | 958,476 |
| #4 | (treatment) OR (diet) OR (dietary) OR (effect) OR (effectiveness) OR (efficacy) OR (efficiency) OR (response) OR (affect) OR (management) OR (impact) | 24,548,430 | 62,169,580 | 33,506,094 |
| #5 | (humans) OR (patients) OR (adults) OR (participants) | 25,623,935 | 41,261,128 | 16,673,202 |
| #6 | #1 AND #2 AND #3 AND #4 AND #5 | 447 | 2679 | 1230 |

**Table S2.** Results of the quality assessment of randomised studies using the CASP Randomised Controlled Trial Standard Checklist [12] - Sections A and B

| Authors, year | Section A: Is the basic study design valid for a randomised controlled trial? | | | Section B: Was the study methodologically sound? | | | | |
|---|---|---|---|---|---|---|---|---|
| | Did the study address a clearly focused research question? | Was the assignment of participants to interventions randomised? | Were all participants who entered the study accounted for at its conclusion? | Were the participants 'blind' to intervention they were given? | Were the investigators 'blind' to the intervention they were giving to participants? | Were the people assessing/analysing outcome/s 'blinded'? | Were the study groups similar at the start of the randomised controlled trial? | Apart from the experimental intervention, did each study group receive the same level of care (that is, were they treated equally)? |
| Böhn et al., 2015 | Yes | Yes | No | Yes | No | No | Yes | Yes |
| Eswaran et al., 2017 | Yes | Yes | No | Yes | No | Yes | Yes | Yes |
| Liu et al., 2024 | Yes | Yes | Yes | Yes | Yes | Yes | Yes | Yes |
| Ostrowska et al., 2021 | Yes | Yes | Yes | No | No | No | Yes | Yes |
| Patcharatrakul et al., 2019 | Yes | Yes | Yes | Yes | No | No | Yes | Yes |
| Rej et al., 2022 | Can't tell | Yes | Yes | No | No | Can't tell | Yes | Yes |
| Russo et al., 2022 | Yes | Yes | Can't tell | Yes | No | No | Yes | Can't tell |
| Tunali et al., 2024 | Yes | Yes | Yes | Can't tell | Yes | Yes | Yes | Yes |
| Zahedi et al., 2018 | Yes | Yes | Yes | Yes | No | No | Yes | No |

| | | | | | | | | |
|---|---|---|---|---|---|---|---|---|
| Zhang et al., 2021 | Yes | Yes | Yes | No | No | Yes | Yes | Yes |
| Bodini et al., 2019 | Yes | Yes | Yes | Can’t tell | Yes | Can’t tell | Can’t tell | Yes |
| Cox et al. 2020 | Yes | Yes | No | Yes | No | No | Can’t tell | Yes |
| Pedersen et al., 2017 | Yes | Yes | No | No | No | No | Yes | No |
| Rivière et al., 2021 | Yes | Yes | Can’t tell | No | No | No | Yes | Yes |

Table S2. *Continuation* - Sections C and D

| Authors, year | Section C: What are the results? | | | Section D: Will the results help locally? | | Overall assessment |
|---|---|---|---|---|---|---|
| | Were the effects of intervention reported comprehensively? | Was the precision of the estimate of the intervention or treatment effect reported? | Do the benefits of the experimental intervention outweigh the harms and costs? | Can the results be applied to your local population/in your context? | Would the experimental intervention provide greater value to the people in your care than any of the existing interventions? | |
| Böhn et al., 2015 | Yes | Yes | Yes | Yes | Can't tell | Major limitations |
| Eswaran et al., 2017 | No | Yes | Yes | Yes | Can't tell | Major limitations |
| Liu et al., 2024 | Yes | Yes | Yes | Can't tell | Can't tell | Minor limitations |
| Ostrowska et al., 2021 | Yes | Yes | Can't tell | Yes | Can't tell | Major limitations |
| Patcharatrakul et al., 2019 | Yes | Yes | Yes | Can't tell | Can't tell | Minor limitations |
| Rej et al., 2022 | Yes | Yes | Can't tell | Yes | Can't tell | Major limitations |
| Russo et al., 2022 | Yes | Yes | Yes | Can't tell | Can't tell | Minor limitations |
| Tunali et al., 2024 | Yes | Yes | Yes | Yes | Can't tell | Minor limitations |
| Zahedi et al., 2018 | Yes | Yes | Yes | Yes | Can't tell | Major limitations |
| Zhang et al., 2021 | Can't tell | Yes | Yes | Can't tell | Can't tell | Minor limitations |
| Bodini et al., 2019 | Can't tell | Can't tell | Yes | Yes | Can't tell | Minor limitations |
| Cox et al. 2020 | No | No | Yes | Yes | Can't tell | Major limitations |
| Pedersen et al., 2017 | Can't tell | Can't tell | Yes | Yes | Can't tell | Major limitations |

| | | | | | | |
|---|---|---|---|---|---|---|
| Rivière et al., 2021 | Yes | Yes | Can’t tell | Yes | Can’t tell | Major limitations |
